# The Pathology and Treatment of Venereal Diseases; Etc.

**Published:** 1861-10

**Authors:** 


The Pathology and Treatment of Venereal Diseases; including the Results of Recent Investigations on the Subject. By Freeman J. Bumstead, M. D., Lecturer on Venereal Diseases, at the College of Physicians and Surgeons, New York; Surgeon to St. Luke's Hospital; Assistant-Surgeon to the New York Eye Infirmary. Philadelphia: Blanchard & Lea. 1861.

Through W. B. Keen, 148 Lake Street, we are also placed in possession of this volume, whose receipt only, at this time, do we propose to acknowledge,—reserving for our succeeding number a suitable notice.

